# Comparison of outcome of Ponseti method with traditional clubfoot treatment in children up to five years of age at tertiary care hospital

**DOI:** 10.12669/pjms.38.6.5519

**Published:** 2022

**Authors:** Ayesha Yaqeen, Hafiza Sidra, Muhammad Abdullah Ijaz, Muhammad Muneeb Ijaz

**Affiliations:** 1 Dr. Ayesha Yaqeen, MBBS, Department of Orthopedics, Nishtar Medical University & Hospital, Multan, Pakistan; 2 Dr. Hafiza Sidra, MBBS, Department of Orthopedics, Nishtar Medical University & Hospital, Multan, Pakistan; 3 Dr. Muhammad Abdullah Ijaz, MBBS. Department of Community Medicine, Multan Medical & Dental College, Multan, Pakistan; 4 Dr. Muhammad Muneeb Ijaz, DPT, Department of Orthopedics, Nishtar Medical University & Hospital, Multan, Pakistan

**Keywords:** Clubfoot, Surgical treatment, Ponseti, Talar flattening, Outcomes

## Abstract

**Objectives:**

To compare the outcomes of Ponseti treatment with the traditional treatment method for clubfoot.

**Methods:**

A cross-sectional comparative study was conducted at the orthopedic department of Nishtar Medical Hospital & University Multan for one year. The study included 40 children (29 clubfeet) treated with conventional treatment (pre-Ponseti group) who were compared with 55 Ponseti-treated children (72 clubfeet) (Ponseti group). All children were aged under five years. The traditional treatment involved casting and surgery (if required). All the participants were evaluated by a single orthopedic surgeon. The questionnaire was administered to the parents to collect relevant data. X-ray studies were conducted of all feet and patients’ records were checked for surgical history.

**Results:**

Children in the pre-Ponseti group had a significantly higher number of surgeries (54) than those in Ponseti group eight. According to the reports of the parents, children in Ponseti group had significantly better motion in the ankle, lesser pain, and higher satisfaction (p<0.05.whereas, the pre-Ponseti group had a higher incidence of moderate or severe talar flattening rate (p=0.01).

**Conclusion:**

Ponseti treatment is better than earlier treatment in terms of lesser need of surgeries, higher flexibility of ankle or foot, and lower presence of X-ray guided talar flattening.

## INTRODUCTION

Idiopathic clubfoot is a genetic structural deformity resulting as a consequence of multiple causes in otherwise normal children. The therapeutic strategies aim to a plantigrade and a pain-free foot with structural integrity. Earlier on, most of the children needed surgical interventions; however, from the last twenty years, the Ponseti treatment is perceived as a standard treatment of clubfoot across the world.^1^-^3^

Traditionally, clubfoot is treated clinically but physiotherapy has also been proven effective even in non-idiopathic and complicated cases.^4^ The survey data suggests the drop in conduction of extensive surgeries for treating clubfoot from 70% in 1996 to only about 10% in 2006 in the USA.^5^ However, it is surprising to find out that only a few studies have been conducted in Pakistan that compared Ponseti treatment with earlier treatment methods.

To our knowledge, Ponseti treatment was formally adopted by many hospitals in Pakistan in 2012 and the clinical trials found it effective.^6^ Before the Ponseti method, children were treated with distinct methods of serial casting followed by surgery, if required. In such surgical treated feet, pain, stiffness, and persistence of deformity are reported.^7^,^8^ The purpose of the present study was to compare the Ponseti method with previous treatment methods in terms of number and types of required surgeries; deformity and flexibility of ankle and foot; resolution of pain, and x-ray reported talar flattening.

## METHODS

A cross-sectional comparative study was conducted at the orthopedic department of Nishtar Medical University & hospital Multan. Records of all the children who visited the hospital between 2015-2020 with an idiopathic club foot and were aged under five years during their treatment were included in the study. However, those with a neurological club foot or those who didn’t complete the treatment were excluded from the study. The analysis of records and follow-up of the treated children was carried out taking ethical committee approval ref no.29/108 on dated 08.05.2020 for 1 year from 8^th^ June 2020 to 8^th^ June 2021. The record data collected were divided into two groups: pre-Ponseti and Ponseti group. In the pre-Ponseti group, records of 40 children (29 club feet) were found and in the Ponseti group, 55 children (72 club feet) were analyzed. Children in the pre-Ponseti group underwent traditional treatment by regular replacement of above-the-knee casts, either of Plaster of Paris (10 feet) or synthetic soft tissue (19 feet) for three months (2-4 months), followed by surgery if required. To prevent relapse, unilateral orthosis was recommended for all such patients for around 18 months. Primary surgery or later surgeries due to relapse were conducted on the indication orthopedic surgeon. In the Ponseti group, all children also underwent changing of above-the-knee casts, either of Plaster of Paris (22 feet) or synthetic soft cast (52 feet). If required, tenotomy of the Achilles tendon was done. To prevent relapse, a brace was kept for four years. The brace was either a traditional bilateral foot abduction brace (59%) or a special unilateral above-the-knee brace (41%). Seventy percent of these children used the brace for four years, six hours daily; 20% for two years, and 10% stopped using before two years. Here too, surgery in all patients was recommended by the surgeon based on his experience. All the children in the study started receiving their respective treatments in the first week of their life except one case of a delayed diagnosis (seven weeks) in the Ponseti group.

While recording the surgical data for comparison, tenotomy of the Achilles tendon conducted in the Ponseti group was not considered as surgery. Whereas, tendon transfers, lengthening, and other tenotomies were grouped as “minor surgeries”. Extensive surgeries included osteotomies, posteromedial release, and posterior release. Moreover, a goniometer was used to measure foot adduction, intermalleolar axis, and range of motion. The cases with unilateral clubfeet were compared with other normal feet for leg length difference on standing, calf circumference, and foot length. The level of pain experienced by the child, function of feet, and satisfaction were evaluated by the child’s through two questionnaires: the Functional Rating System for clubfoot^9^ and the Disease-Specific Instrument for clubfoot.^10^ The Function Rating System had a maximum of 100 scores which were divided into questions related to a child’s satisfaction (20 points), pain (30 points), and function (20 points). Additionally, some scores (10 points) are also allocated to examiner’s evaluation of the position of heel; 10 points to all together evaluation of inversion-eversion foot movement, varus-vagus heel movement, and flexibility of foot; and 10 points for the evaluation of gait pattern. The Disease-Specific Instrument involves 10 questions related to pain, satisfaction, and function. All 10 questions were responded to on a scale of 0 (worst) to 100 (best). Follow-up examination also included radiological examination, of lateral and anteroposterior sides, of both feet. The lateral views were ranked from 0 (physiological curve) to 3 (total flattening) and compared between two groups.

SPSS (version 18.0) was used for statistical evaluation. Generalized estimation equations (GEE) with a log-link and a Poisson distribution were used for the comparison of several surgeries between two groups. The continuous data were analyzed through a mixed model that allowed adjustment for repeated measures for patients. The radiological variables were evaluated through the GEE model for binary data. P-value less than 0.05 for any variable was considered statistically significant.

## RESULTS

The data collected on the operative history of the participants were divided into minor and extensive surgeries that are presented in Table-I. Both minor surgeries and extensive surgeries were significantly lesser in the Ponseti group than in the other evaluated group. Children in the pre-Ponseti group most frequently underwent posteromedial release (34 feet) followed by posterior release (21 feet). Whereas in the Ponseti group, tendon lengthening (8 feet) was the most frequent surgery followed by open tenotomies (6 feet) and posteromedial release (6 feet). In terms of flexibility, dorsal and plantar flexion was significantly improved in the Ponseti group than in the pre-Ponseti group (15º vs 17º and 23º vs 28º, respectively). However, the external rotation of the ankle or foot was almost similar. The intermalleolar axis was decreased in the children from the pre-Ponseti group whereas the intermalleolar axis in the Ponseti treated feet was similar to that of healthy feet (Table-II).

**Table-I:** Surgical data of the study groups.

	Pre-Ponseti group (N=29 feet)	Ponseti group (N=72 feet)	P-value
Minor surgery	20	17	.02
Open tenotomies	4	6	
Tendon lengthening	10	8	
Tibialis anterior transfer	6	3	
Extensive surgery	65	11	<.01
Posterior release	21	3	
Postero-medial release	34	6	
Osteotomies	10	1	
Overall Surgeries	85	28	<0.01

**Table-II:** Clinical Outcomes of the Procedures.

	Pre-Ponseti group (N=29 feet)	Ponseti group (N=72 feet)	Mean difference (95% CI)	P-value
***Flexibility (mean)***				
Dorsal flexion	15º	17º	2.7 (0.8 - 4.5)	0.004
Plantar flexion	23º	28º	3.1 (0.7 - 4.9)	0.006
External rotation	36º	37º	0.3(0.1 - 1.9)	0.72
***Appearance (mean)***				
Foot adduction	3º	3º	0.1 (-1.1 - 1.3)	0.9
Intermalleolar axis	20º	23º	2.9 (1.0 - 4.2)	0.001
foot length difference	13mm	11mm	1.1 (0.1 - 0.6)	0.5
leg length difference	4mm	1mm	0.3 (-0.0 to 0.3)	0.07
calf circumference difference	24 mm	17mm	0.8 (0.4 to 1.2)	0.001

The treatment outcome was scored by parents both as per Functional Rating System (Table-III) and Disease-Specific Instrument for clubfoot (Table-IV). Among the functional outcomes, the highest difference between the groups was found in terms of pain experienced. In the pre-Ponseti group, children in the unilateral group experienced lesser pain than those in bilateral clubfeet. The score difference was also significant between two groups in the subcategories of “satisfaction”, “function”, “varus-valgus flexibility”, and “gait” (p<0.05). Within the Ponseti group, however, no significant difference was found between those with unilateral clubfeet and bilateral clubfeet in terms of parents’ reported outcome. The Pre-Ponseti group had a significantly higher number of clubfeet with mild and moderate talar flattening (Table-V).

**Table-III:** Scoring based on Functional Rating System for clubfoot.

	Pre-Ponseti group (N=29 feet)	Ponseti group (N=72 feet)	Mean difference (95% CI)	P-value
***Parent-reported scores***				
Satisfaction	15	18	2.1 (0.5 - 2.7)	0.01
Function	16	18	1.9 (1.2 - 3.3)	0.01
Pain	23	26	2.9 (1.6 – 4.0)	0.01
***Physical evaluation***				
Heel position (max 10 scores)	8.8	8.4	0.4 (0.9 - 1.3)	0.2
Dorsal flection (max 5 scores)	3.1	3.5	0.4 (0.1 - 0.7)	0.04
Varus-valgus (max 3 scores)	2.5	2.7	0.4 (0 - 0.5)	0.04
Inversion-eversion (max 2 scores)	2.1	2.1	.001 (-)	0.9
Gait (max 10 scores)	8.3	8.9	0.5 (0.2 - 0.9)	0.001
Total score (max 100 scores)	78.8	87.6	7.9 (4.2 to 8.9)	0.001

**Table-IV:** Scoring based on Diseases Specific Instrument for clubfoot.

	Pre-Ponseti group (N=29 feet)	Ponseti group (N=72 feet)	P-value
***Satisfaction***			
Foot status	7.0	7.9	0.01
Foot appearance	7.2	8.0	0.03
Extend of teasing	9.4	9.8	0.001
Ease in finding an appropriate shoe	6.2	7.6	0.01
Ease in finding favorite shoes	6.9	8.1	0.02
***Pain and Function***			
Pain	2.3	5.3	<0.01
Walking limitations	7.6	8.8	<0.01
Difficulty limitations	6.4	7.7	0.002
Pain while doing heavy exercise	6.3	7.4	0.04
Pain while doing moderate exercise	7.3	8.5	<0.001
Total score (0-100 pts.)	66.6	79.1	

**Table-V:** Comparison of Talar flattening in two study groups.

	Pre-Ponseti group (N=29 feet)	Ponseti group (N=72 feet)	P-value
Normal	3 (10.3%)	12 (16.6%)	0.04
Mild	13 (44.8%)	43 (57%)	
Moderate	11 (37.9%)	13 (18%)	
Severe	2 (6.8%)	4 (5.5%)	

## DISCUSSION

The study showed that children had better outcomes following Ponseti treatment. The children in the Ponseti group reported improvement in dorsal and plantar flexion. However, no significant betterment in foot abduction and external rotation was observed. Moreover, the severity of talar flattening reduced after Ponseti treatment. This is in line with results reported in previous studies.^11^,^12^

Our study has reported that 11 Ponseti treated feet had to undergo extensive surgeries due to various reasons. One of the previous related studies reported 10% of patients required extensive surgery as the disease relapsed in them.^11^ Although few studies found a relatively lower surgery rate due to relapse following Ponseti treatment, these studies had a shorter follow-up time.^13^ One of the major aims of Ponseti treatment is to improve ankle movement in patients with clubfeet. Our study has reported better dorsal flexion of the ankle when compared with other studies.^12^,^14^ Our study also found out that children in the pre-Ponseti group had a reduced intermalleolar axis than those in the Ponseti group. Similarly, these children had larger variation in calf circumference and higher incidence of leg length variation than those in the Ponseti group. However, Smith et al. found no such discrepancy in their study.^14^

It is, no doubt, challenging to assess the outcomes of clubfoot treatment. The treatment can’t be approved unless the physician, patient, and parents all are equally satisfied. Therefore, we utilized two questionnaires. The Function Rating System for club foot is the most frequently utilized rating scheme for the assessment of long-term outcomes of clubfoot treatment.^15^,^16^ It was for the first time adopted by Laaveg and Ponseti on elder clubfeet patients, between 10 to 27 years. The study reported a score of 87.5 out of 100 in the Ponseti group which is comparable to our score of 86.5.^17^ Under the same method, the results reported in our study for the pre-Ponseti group are better than those found by Dobbs et al.^18^ However, the score of the pre- Ponseti group is lower when compared with the results of Svehlik et al.^19^

The Disease-Specific Instrument for clubfoot is designed for parents. The questioner was for the time used for operated clubfeet^20^ but was later utilized for Ponseti treated clubfeet as well.^21^ In our study, the responses of parents in the pre-Ponseti group summed up a score of 66.6 that complies with the results of Roye’s work (68.6)^20^ but is inferior to the results of Dietz study (75 scores)^10^ who evaluated the surgically treated patients. Although, most of the questions were responded to by parents children also contributed to certain responses such as to extend of teasing. The questionnaire was kept specific for clubfeet and excluded health-related questions since children with idiopathic clubfeet are generally healthy.

We also investigated the difference in talar flattening between the two evaluated groups. The results found that in the Ponseti group 16.6% had no talar flattening while 5% had severe talar flattering. These results are not similar with the results of Hutchin et al. who reported absence of talar flattening in 26% of patients and only 1.5% suffered greatly from the disorder.^22^

### Limitations of the study

Although, the study analyzed five years of data, the sample size still appears to be small. Therefore, a multi-center comparative study is recommended while avoiding the influence of confounding variables.

## CONCLUSION

Ponseti treatment is better than earlier treatment strategy in terms of lesser need of surgeries, higher flexibility of ankle or foot, and lower presence of X-ray guided talar flattening.

### Authors’ Contribution:

**AY, HS, MMI:** Conceived, designed and did statistical analysis & editing of manuscript.

**HS, MAI, MMI:** Did data collection and manuscript writing.

**AY, MAI:** Did review and final approval of manuscript.

**MMI, HS:** Responsible for accountability & integrity of the study.

## References

[1] Al-Mohrej OA, Alshaalan FN, Alhussainan TS (2021). Is the modified Ponseti method effective in treating atypical and complex clubfoot?A systematic review. Int Orthop.

[2] Agnihotri A, Chand S, Mehtani A, Sud A, Sinha S, Kumar A (2021). Changes in the Foot After Two Years of Deformity Correction in Neglected Clubfeet Treated With Modified Ponseti Technique. Cureus.

[3] Mukerjee KB (2004). Long-term comparative results in patients with congenital clubfoot treated with two different protocols. J Bone Joint Surg Am.

[4] Dunkley M, Gelfer Y, Jackson D, Parnell E, Armstong J, Rafter C (2015). Mid-term results of a physiotherapist-led Ponseti service for the management of non-idiopathic and idiopathic clubfoot. J Children's Orthop.

[5] Zionts LE, Zhao G, Hitchcock K, Maewal J, Ebramzadeh E (2010). Has the rate of extensive surgery to treat idiopathic clubfoot declined in the United States?. J Bone Joint Surg Am.

[6] Rehman MU, Salim M, Ahmed M (2018). Management of Club Foot by Ponseti Method:Our experience. Pak Armed Forces Med J.

[7] Chotigavanichaya C, Wongchareonwatana J, Saelim C, Ariyawatkul T, Kaewpornsawan K, Eamsobhana P (2019). Comparison of ponseti method versus surgical treatment in congenital idiopathic clubfoot:A 5-year follow up study. Int J Orthop Sci.

[8] Adegbehingbe OO, Adetiloye AJ, Adewole L, Ajodo DU, Bello N, Esan O (2017). Ponseti method treatment of neglected idiopathic clubfoot:Preliminary results of a multi-center study in Nigeria. World J Orthop.

[9] Lehman W, Atar D, Grant A, Strongwater A (1994). Functional rating system for evaluation of long-term results of clubfoot surgery. The Clubfoot:Springer.

[10] Dietz FR, Tyler MC, Leary KS, Damiano PC (2009). Evaluation of a disease-specific instrument for idiopathic clubfoot outcome. Clin Orthop Related Res.

[11] Halanski MA, Davison JE, Huang J-C, Walker CG, Walsh SJ, Crawford HA (2010). Ponseti method compared with surgical treatment of clubfoot:A prospective comparison. J Bone Joint Surg Am.

[12] Smith PA, Kuo KN, Graf AN, Krzak J, Flanagan A, Hassani S (2014). Long-term results of comprehensive clubfoot release versus the Ponseti method:which is better?. Clin Orthop Related Res.

[13] Morcuende JA, Dolan LA, Dietz FR, Ponseti IV (2004). Radical reduction in the rate of extensive corrective surgery for clubfoot using the Ponseti method. Pediatrics.

[14] Chand S, Mehtani A, Sud A, Prakash J, Sinha A, Agnihotri A (2018). Relapse following use of Ponseti method in idiopathic clubfoot. J Children's Orthop.

[15] Agarwal A, Gupta S, Agarwal S (2020). Nwdps protocol-A simple functional outcome assessment tool for clubfoot correction and a review of literature. J Clin Orthop Trauma.

[16] Zionts LE, Ebramzadeh E, Morgan RD, Sangiorgio SN (2018). Sixty years on:Ponseti method for clubfoot treatment produces high satisfaction despite inherent tendency to relapse. J Bone Joint Surg Am.

[17] Laaveg SJ, Ponseti IV (1980). Long-term results of treatment of congenital club foot. J Bone Joint Surg Am Vol.

[18] Dobbs MB, Nunley R, Schoenecker PL (2006). Long-term follow-up of patients with clubfeet treated with extensive soft-tissue release. J Bone Joint Surg Am.

[19] Svehlik M, Floh U, Steinwender G, Sperl M, Novak M, Kraus T (2017). Ponseti method is superior to surgical treatment in clubfoot - Long-term, randomized, prospective trial. Gait Posture.

[20] Roye BD, Vitale MG, Gelijns AC, Roye DP (2001). Patient-based outcomes after clubfoot surgery. J Pediat Orthop.

[21] Gelfer Y, Hughes KP, Fontalis A, Wientroub S, Eastwood DM (2020). A systematic review of reported outcomes following Ponseti correction of idiopathic club foot. Bone Joint Open.

[22] Hutchins P, Foster B, Paterson D, Cole E (1985). Long-term results of early surgical release in club feet. J Bone Joint Surg Brit Vol.

